# Genetic Parameter Estimation and Whole Sequencing Analysis of the Genetic Architecture of Chicken Keel Bending

**DOI:** 10.3389/fgene.2022.833132

**Published:** 2022-03-23

**Authors:** Zhihao Zhang, Weifang Yang, Tao Zhu, Liang Wang, Xiaoyu Zhao, Guoqiang Zhao, Lujiang Qu, Yaxiong Jia

**Affiliations:** ^1^ Institute of Animal Sciences, Chinese Academy of Agricultural Science, Beijing, China; ^2^ Beijing General Station of Animal Husbandry, Beijing, China; ^3^ State Key Laboratory of Animal Nutrition, Department of Animal Genetics and Breeding, National Engineering Laboratory for Animal Breeding, College of Animal Science and Technology, China Agricultural University, Beijing, China; ^4^ Hebei Dawu Poultry Breeding Co., Ltd., Hebei, China

**Keywords:** chicken, keel bend, genetic parameters, pool-seq, candidate gene

## Abstract

Bone health is particularly important for high-yielding commercial layer chickens. The keel of poultry is an extension of the abdomen side of the sternum along the sagittal plane and is one of the most important bones. In this study, the keel phenotype of White Leghorns laying hen flocks showed significant individual differences. To clarify its genetic mechanism, we first estimated the heritability of keel bend (KB) in White Leghorn, recorded the production performance of the chicken flock, examined the blood biochemical indexes and bone quality in KB and keel normal (KN) chickens, and performed whole-genome pooled sequencing in KB and KN chickens. We then performed selection elimination analysis to determine the genomic regions that may affect the keel phenotypes. The results show that KB is a medium heritability trait. We found that cage height had a significant effect on the KB (*p* < 0.01). At 48 weeks, there were significant differences in the number of eggs, the number of normal eggs, and eggshell strength (*p* < 0.05). The content of parathyroid hormone was lower (*p* < 0.01) and that of calcitonin was higher (*p* < 0.01) in KB chickens than in KN chickens. The differences in bone mineral density, bone strength, and bone cortical thickness of the humerus and femur were extremely significant (*p* < 0.01), with all being lower in KB chickens than in KN chickens. In addition, the bones of KB chickens contained more fat organization. A total of 128 genes were identified in selective sweep regions. We identified 10 important candidate genes: *ACP5, WNT1, NFIX, CNN1, CALR, FKBP11, TRAPPC5, MAP2K7, RELA,* and *ENSGALG00000047166*. Among the significantly enriched Kyoto Encyclopedia of Genes and Genomes pathways found, we identifed two bone-related pathways, one involving “osteoclast differentiation” and the other the “MAPK signaling pathway.” These results may help us better understand the molecular mechanism of bone traits in chickens and other birds and provide new insights for the genetic breeding of chickens.

## Introduction

Laying hens provide eggs and meat for human use and are one of the most important poultry in the global breeding industry. With the significant increase in the demand for poultry eggs (Mueller et al., 2018), commercial layer strains have reached very high egg production levels (Toscano et al., 2020). To form an eggshell of the quality expected by consumers, each egg needs approximately 2–3 g of calcium (Roberts, 2004; Kebreab et al., 2009). The calcium required for the synthesis of eggshells mainly comes from feed (approximately 65%) and bone absorption (about 35%; Mueller et al., 1964; Buss and Guyer, 1984). Bone is the main repository of calcium (Li et al., 2017). When the calcium supply in the feed is insufficient, the endogenous calcium in the bones undergoes reabsorption to meet the needs of daily egg production (Nys and Le Roy, 2018). Therefore, bone health is particularly important for laying hens.

The keel of poultry is an extension of the abdomen side of the sternum along the sagittal plane and is one of the most important bones of poultry. It is an important structure responsible for the flight ability of birds and it plays an important role in breathing (Claessens, 2009; Wei et al., 2020). The keel of poultry is usually the first point of contact when a collision occurs, so it is easily damaged (Donaldson et al., 2012), which is known as keel bone damage (KBD). KBD mainly includes two types, keel fractures and keel bends (KB), which are mainly manifested as bending, offset, or creasing of the keel part or whole. It can also be called keel deformation, keel deviation, and keel fracture, which are all symptoms of KBD (Casey-Trott et al., 2015). It is generally believed that the greatest risk of keel fractures comes from collision between laying hens and their cage and the weakening of keel strength that results (Casey-Trott et al., 2015). KB, defined as a keel with an abnormal shape caused by non-fracture, is a less-mentioned type of keel damage (Casey-Trott et al., 2015). For a normal keel, the front of the keel ridge should follow a straight line; deformation causes a deviation from this straight line. Deviations can be in the vertical or horizontal direction, creating an S-shaped appearance, protrusions, recesses, or other shapes. The terms used to describe such deviations include “S-shaped,” ‘twisted,’ and ‘curved’ (Lay et al., 2011). Curvature may be caused by the destruction of the keel periosteum surface rather than being a direct result of fracture or impact injury (Fleming et al., 2004). In contrast to fractures, the development of KB takes a period of time, which is the result of the body’s response to normal load and pressure, that is, the process of bone remodeling (Riber et al., 2018).

KB affects the physical condition of laying hens, resulting in acute or chronic physiological stimulation of sick laying hens (Nasr M. A. F. et al., 2012; 2013). This is not only an animal welfare issue, but also affects the health status and production performance of laying hens (Nasr M. et al., 2012; Gebhardt-Henrich and Frohlich, 2015) which then affects the economic benefits of the laying hen industry. KB is a trait that is easy to identify during the breeding process. Moreover, studies have reported that the bone characteristics of laying hens can be moderately to highly inherited (Bishop et al., 2000). Because of the high heritability of chicken bone traits and the increasing number of bone problems, research on chicken bone traits at the gene level is gradually increasing. In a recent study, 21 candidate genes that may regulate chicken bone growth and development were identified (Li et al., 2021), namely *LRCH1, RB1, FNDC3A, MLNR, CAB39L, FOX O 1, LHFP, TRPC4, POSTN, SMAD9, RBPJ, PPARGC1A, SLIT2, NCAPG, NKX3-2, CPZ, SPOP, NGFR, SOST, ZNF652*, and *HOXB3*. Previous studies reported genes affecting the tibia and femur of laying hens (Guo et al., 2020), including *HTR2A, LPAR6, CAB39L, TRPC4, WNT9A, SPOP, NGFR, GIP,* and *HOXB3*. Furthermore, candidate genes associated with chicken osteoporosis have also been identified (Guo et al., 2017), including *RANKL, ADAMTS*, and *SOST*.

Compared with other bones of chicken, the keel is completely ossified later (Buckner et al., 1949). Therefore, some areas of the keel remain cartilaginous for a long time (Casey-Trott et al., 2017). Hartmann and Tabin (2000) found that *WNT-5a*, *WNT-5b*, and *WNT-4* genes are expressed in the chondrogenic region of chicken limb. Their experiment also shows that an endogenous (Hartmann and Tabin, 2000). The Wnt signal does indeed function to promote chondrogenic differentiation. At the same time, some research findings suggest a functional role for Wnt signaling throughout embryonic cartilage development (Daumer et al., 2004). In addition, MAPK signaling pathway-related genes *RAC2, MAP3K1, PRKCB, FLNB, IL1R1, PTPN7, RPS6KA, MAP3K6, GNA12*, and *HSPA8* play an important role in chicken tibial dyschondroplasia (Jahejo et al., 2019). Therefore, it is important to explore the genetic basis of KB in poultry.

In this study, we estimated the genetic parameters of KB in White Leghorn laying hens and analyzed the relationship between KB and production performance. We divided chickens into two groups: those with a normal keel (“keel normal”; KN) and those with KB. The blood biochemical indices related to bone metabolism and the differences in the humerus, femur, and keel between the two groups were compared. Finally, we performed whole-genome pooled sequencing (Pool-Seq) on line White Leghorn chickens with an obvious KB or KN. By screening the genomic regions that experienced selective sweeps, genes related to the keel were identified, and the underlying molecular genetic mechanisms were explored.

## Materials and Methods

### Animals and Data Collection

The laying hens used in the experiment were commercial White Leghorns laying hen strains from a Chinese laying hen breeding company. The total number of laying hens was 1,600. All hens were raised in three-layer H-shaped cages in the same chicken house with closed and mechanical ventilation. Single cage feeding was used. Each single cage was 40 cm long, 45 cm wide, 45 cm high at the front, and 38 cm high at the back. The feeding density was approximately six animals per square meter according to the calculation of a single layer of a single cage. During the experiment, the same management method and feeding system were used throughout. The KB level of each hen in the experimental flock was determined when the chickens were 30 and 46 weeks old, and the cage height was recorded.

**FIGURE 1:**
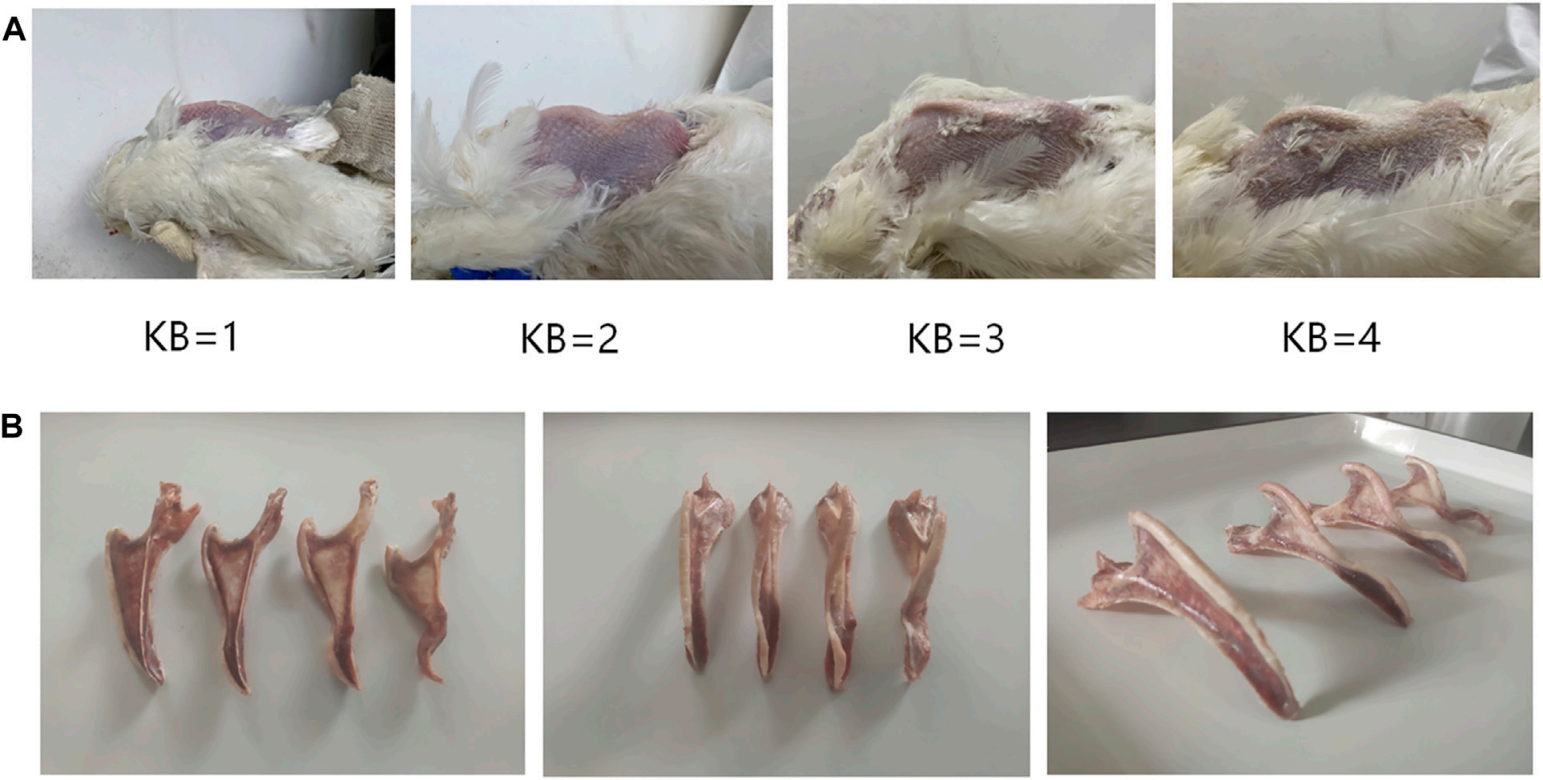
**(A)** Reference images of KB levels in White Leghorn laying hens **(B)** Anatomical diagram of KB levels in White Leghorn laying hens.

The level of KB was determined using manual palpation. During palpation, the wings of the chicken were held from the wing root in one hand of the palpator to expose the chicken’s abdomen. Then, the index finger and thumb of the palpator’s other hand were used to examine the ventral and lateral edges of the keel to determine the level of KB. To improve the efficiency of evaluation, we developed an easy and effective method for determining the level of KB. The grading criteria of our method for evaluating KB levels are summarized in Table 1. In summary, KBs can be categorized into one of four levels according to the degree of the bend: normal, slight, moderate, or severe, recorded as 1, 2, 3, and 4, respectively (Figure 1A). Examples of the different levels are shown in Figure 1B. To ensure consistency in assessment, only one palpator was used, and the palpator assessed 100 chickens as a training exercise prior to data gathering. These chickens were assessed many times until the consistency rate of the palpation results of the last two evaluations reached about 90%. In order to study the relationship between KB and production performance in laying hens, we counted the total number of eggs and the total number of “normal” eggs (i.e., excluding those that had a double yolk, soft shell, unsmooth shell, broken shell, or were deformed) of all experimental laying hens from the beginning of laying to 48 weeks (336 days) old. At 48 weeks, we collected the eggs produced by all experimental laying hens on a given day and measured their weight and eggshell strength. To measure the eggshell strength, the egg is placed in the upward position and an eggshell strength tester (HAD-A1, Beijing Hengaode Instrument Co., Ltd., Beijing, China) was used for measurement.

**TABLE 1:** Classification of KB severity in White Leghorn chickens.

Level^a^	Description of KB^b^
1	Normal: the keel is streamlined and there is no dent or deviation
2	Slight: the keel is slightly sunken or deviated (visual inspection means that it is easy to make mistakes, thus, this needs to be confirmed by palpation)
3	Moderate: the keel is obviously sunken or deviated (palpation results are consistent with the visual results)
4	Severe: the keel is severely sunken or deviated (palpation results are more serious than the visual results)

a Level: Numbers 1, 2, 3, and 4 indicate the degree of keel bend.

b KB, keel bend.

### Genetic Parameters Estimation

In this study, the DMU (v6) software package was used to estimate the components of genetic variance (Madsen et al., 2006). The rjmc module was used to analyze the KB data of the 30- and 46-week-old chickens. The calculation method used was the Bayesian method based on Gibbs sampling (Ødegård et al., 2010). In this study, the heritability of keel traits was calculated using a univariate animal model. The variance component was estimated using the following formula:

y=Xb+Za+e

where y is the phenotypic value of chicken KB; X and Z are the correlation matrices of the fixed effect and the random additive effect, respectively; b is the fixed effect vector, including cage height; a is the random additive effect vector; and e represents the random residuals.

### Blood Biochemical Indices Related to Bone Metabolism

At 50 weeks of age, we randomly selected 10 KN chickens and 10 KB chickens from the experimental chicken flock. We collected 5 ml of blood from the wing vein of each hen using vacuum blood collection vessels. The serum levels of calcium, phosphorus, parathyroid hormone (PTH), calcitonin (CT), and 1,25-dihydroxyvitamin D3 were measured. To reduce error, measurements were taken twice and average values were used. After blood collection, the left and right femur, left and right humerus, and keel of the chickens were dissected and collected for subsequent index measurements.

### Bone Mineral Density, Bone Mineral Content, and Bone Strength

The bone mineral density (BMD) and bone mineral content (BMC) of the right femur, right humerus, and keel were measured using a dual-energy X-ray absorptiometry device (XR-36, Norland, United States). After the tests, the samples were stored at −20°C for subsequent testing. After thawing at 15°C for at least 12 h, the right femur and right humerus samples were broken using the animal bone three-point bend test. A Y019 physical tester (TA. XT plus, Stable Micro Systems, United Kingdom) was used to take measurements by placing the plane of the bone sample downward and horizontally on the two supports of the test device. During measurement, the bone sample was gently held in place by hand to avoid it slipping to improve the accuracy of the data. The length and diameter of the bone sample were measured, and after many adjustments, the distance between the brackets was set to 40 mm. The pressure tool applied a constant vertical force and moved vertically to the midpoint of the bone at a speed of 10 mm/min. Finally, the bone was broken and the ultimate force F required to break the bone was recorded in Newtons to obtain the maximum flexural strength.

The data for each index measured in this experiment were analyzed using R (version 4.0.2) software. One-way analysis of variance (ANOVA) and multiple comparisons were used to analyze the differences among hens of the 4 KB levels.The results are expressed as the mean ± SEM. The levels of significance are as follows: *p ≤ 0.05, **p ≤ 0.01, ***p ≤ 0.001.

### Bone Slice

For the left femur and left humerus, we collected samples of around 2 cm in length from their epiphysis and diaphysis. The bone samples were immediately immersed in 4% paraformaldehyde. After fixation for 24 h, decalcification for 2 weeks, paraffin embedding, sectioning, hematoxylin and eosin staining, and scanning with an automatic digital section scanner (KF-PRO-120, Ningbo Jiangfeng Biological Information Technology Co., Ltd., Ningbo City, China) was performed. The diaphysis part was cross cut and the epiphysis part was longitudinally cut. After the cross section of the diaphysis was scanned with a scanner, the bone density thickness of the humeral diaphysis and the femoral diaphysis was obtained by measuring the scanned image of the diaphysis.

### Whole-Genome Sequencing

Laying hens were selected according to KB level. At 52 weeks of age, 36 KN chickens and 48 obvious KB chickens were selected. A 2 ml blood sample was collected from the jugular vein of each individual and stored at −80°C. Genomic DNA was extracted using a genomic DNA extraction kit (DN02, Beijing Aidlab Biotechnologies Co., Ltd., Beijing, China). We then used 1% agarose gel and an ultraviolet spectrophotometer to identify the integrity and purity of the genomic DNA and a TE buffer to adjust the concentration of each DNA sample to 50 ng/uL.

The genomic DNA of the 36 KN chickens was mixed in equal amounts to construct a KN pool. In the same way, the genomic DNA of the 48 KB chickens was mixed in equal amounts to construct a KB pool. Library construction and sequencing were performed at Beijing Biomarker Technologies Co., Ltd. to complete the preparation of the two chicken genomic DNA libraries, and ILLUMINA HISEQ 2500 (Illumina, United States) was used for sequencing. The sequencing depth of each individual was 5x, that is, the sequencing depth of the KN pool was 180x, and the sequencing depth of the KB pool was 240x.

### Quality Control and Data Processing

We obtained the original sequence by sequencing contained low-quality reads with adapters. To ensure the quality of the information analysis, raw reads were filtered to obtain clean reads for subsequent information analysis. Only autosomal markers with clear physical location information were used in the analysis. In this study, fastp (v0.19.4) was used to control the quality of the original data. The main steps of data filtering were as follows: 1) reads with adapters were removed; 2) reads with an N content of more than 10% were filtered; and 3) reads containing more than 50% of the bases with a mass value of less than 10 were removed. After obtaining clean data, we used BWA (v0.7.17) to compare them to the chicken reference genome (GRCg6a). The chicken reference genome sequences were obtained from the NCBI database (https://www.ncbi.nlm.nih.gov/assembly/GCF_000002315.5/). We used GATK (v3.6) for mutation detection to obtain the vcf file.

### Detection of Selective Sweeps

To explore the selection sweep regions related to KB, we adopted a selection elimination analysis based on the population fixation coefficient (*F*_ST_). The population fixation coefficient reflects the heterozygosity level of the population alleles and is used to estimate the difference between the average heterozygosity between subpopulations and the average heterozygosity of the entire population. First, we calculated the F_ST_ value for each single nucleotide polymorphism (SNP) site using a custom python script. Then, we used a 50 kb interval as the sliding F_ST_ window, and the 50% overlap interval as the sliding step size. We calculated the sum of the F_ST_ values of all SNP sites in each window and divided it by the number of SNPs in each window to obtain the average F_ST_ of each window value, using the following formula:

$$F_{\mathrm{ST}}=\left(H_{T}-H_{S}\right)/H_{T}$$

where H_T_ represents the average heterozygosity of the compound population. H_S_ represents the average heterozygosity in the subpopulation. We used the F_ST_ mean of the top 1% window as the threshold. Windows larger than this threshold were regarded as candidate regions for selective sweeps.

### Candidate Genes and Functional Annotation

Gene position information was annotated using BioMart in the Ensembl database (Ensembl Genes 104). Candidate genes were searched in a window with *F*_ST_ values >0.1. To determine the possible functions of genes located in the selective scanning region, we converted chicken genes into human homologous genes, uploaded these homologous genes to Kyoto Encyclopedia of Genes and Genomes (KEGG) orthology based annotation system (KOBAS), and used KOBAS-i (Bu et al., 2021) to perform analysis of gene ontology (GO), the KEGG pathway, and KEGG disease. KOBAS is a web server for gene/protein functional annotation (the annotation module) and functional set enrichment (the enrichment module). Given a set of genes or proteins, it can determine whether a pathway, disease, or GO term shows statistical significance. It provides curated sequences and KEGG pathway knowledge for 5,944 species and GO annotations for 71 popular research species. In KOBAS, Fisher’s exact test was used as the data statistical method, and the Benjamini and Hochberg method was used as the False Discovery Rate correction method.

## Results

### KB Is a Medium Heritability Trait

In this study, we selected approximately 1,600 hens as candidates for phenotypic analysis. According to the DMU instructions, Gibbs sampling method was used to estimate the genetic parameters of KB, and the heritability of 30- and 46-week-old hens was found to be 0.26 and 0.24, respectively (Table 2). Our results show that KB is a heritable trait with medium heritability.

**TABLE 2:** The genetic parameters of KB.

	30-week-old chickens	46-week-old chickens
Add	0.043	0.063
SE^1^	0.005	0.009
Residuals	0.120	0.201
SE^2^	0.006	0.011
h^2^	0.263	0.238

SE^1^, stand error of additive effect. SE^2^, stand error of residuals effect.

### KB Correlates With Cage Height and Production Performance

**FIGURE 2:**
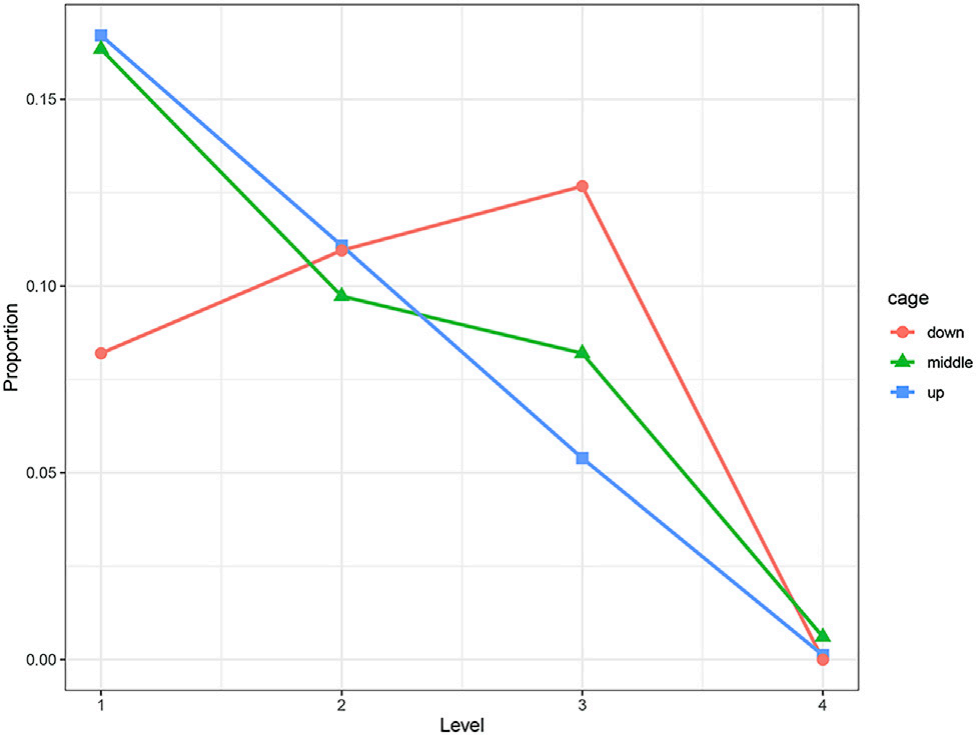
Distribution of KB levels in different cages, where “down” is the first floor, “middle” is the second floor, and “up” is the third floor.

We used the Chi-squared test to examine the effect of cage height on the keel. The results show that cage height has a significant effect on the KB (at 30 weeks of age, *p* < 0.01; at 46 weeks of age, *p* < 0.01). Compared with the middle and upper cages, the proportion of level 1 hens in the bottom cage was significantly less. The proportion of level 2 hens was almost the same, and the proportion of level 3 hens was significantly more. The trends of hens of each level in the middle and upper cages were roughly the same. The proportions of hens in levels 1 to 4 decreased in sequence, and the proportions in each level were similar (Figure 2).

The results of the one-way ANOVA showed that there were significant differences in the total number of eggs, total number of normal eggs, and eggshell strength at 48 weeks (*p* < 0.05), but no significant difference in egg weight (Table 3). The results of multiple comparisons showed that there was no significant differences in egg weight at 48 weeks among the four levels. The total number of eggs and the total number of normal eggs at 48 weeks in level 4 hens were significantly different from that of the other three levels, with the number of eggs and number of normal eggs both lower (Table 3).

**TABLE 3:** Production performances of laying hens at different KB levels.

Item^a^	KB^b^ = 1	KB = 2	KB = 3	KB = 4	*p*-value^e^
EW	58.96^c^ ± 0.13^a^ ^d^	58.86 ± 0.16^a^	58.64 ± 0.18^a^	59.50 ± 0.84^a^	0.486
ES	27.68 ± 0.21^ab^	28.09 ± 0.26^b^	27.04 ± 0.29^a^	30.41 ± 2.78^ab^	0.018*
EN	197.36 ± 0.44^b^	197.54 ± 0.50^b^	196.0 ± 0.56^b^	185.00 ± 4.00^a^	<0.001***
NEN	193.63 ± 0.46^b^	193.97 ± 0.52^b^	192.72 ± 0.58^b^	183.00 ± 4.03^a^	0.011*

a EW, egg weight at week 48; ES, eggshell strength at week 48; EN, total number of eggs from the beginning of laying to 48 weeks of age; NEN, total number of normal eggs (i.e., excluding those that had a double yolk, soft shell, unsmooth shell, broken shell, or were deformed) from the beginning of laying to 48 weeks of age.

b KB, keel bend. 1, 2, 3, and 4 indicate the degree of KB.

c Data represent the mean ± SEM.

d Different letters indicate a significant difference, and the same letters indicate no significant difference (*p* > 0.05).

e *p < 0.05, **p < 0.01, ***p < 0.001, *p* > 0.05.

### Blood Biochemical Indices

**FIGURE 3:**
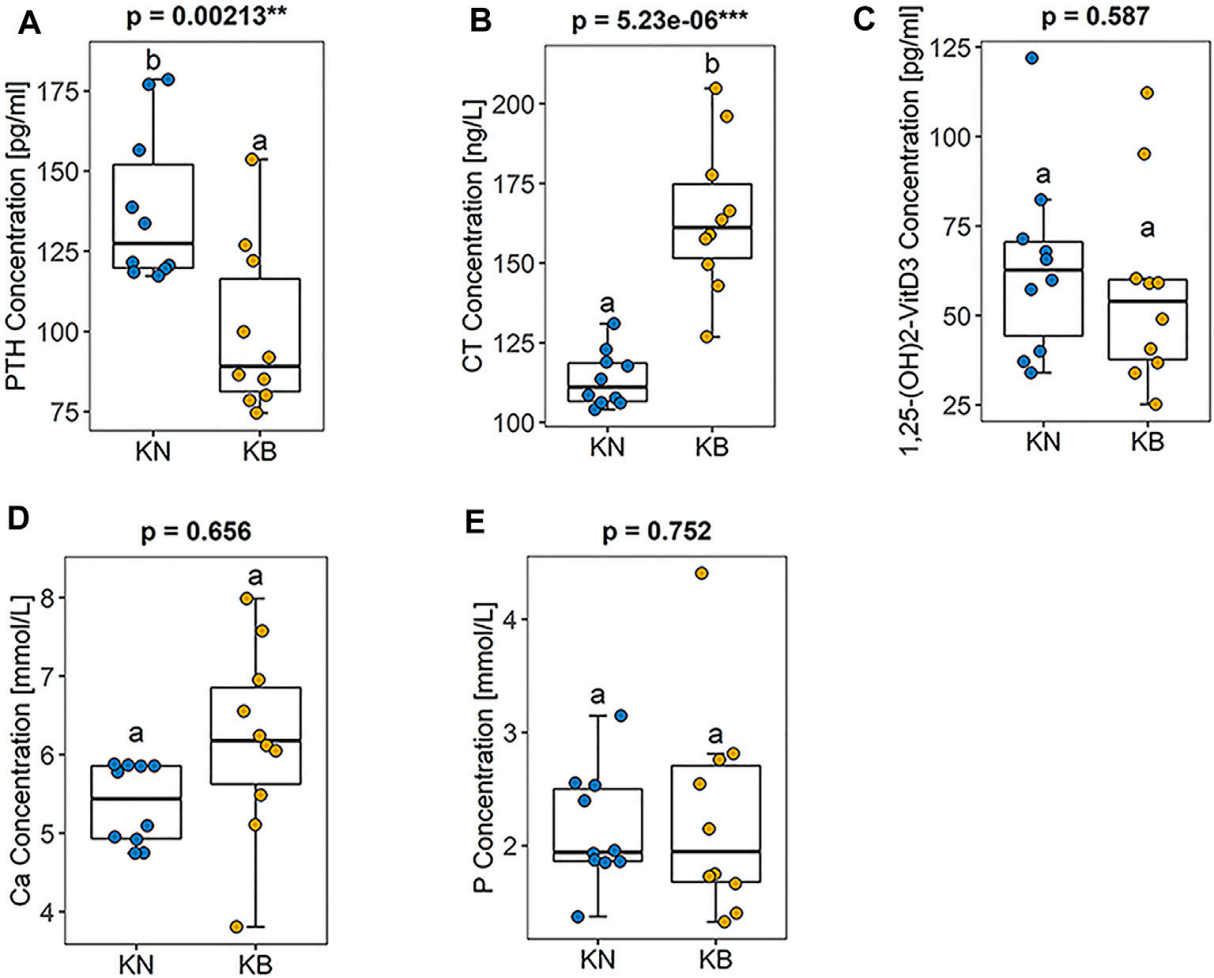
Boxplots of blood biochemical indexes in KN and KB chicken groups **(A)** PTH concentration **(B)** CT concentration **(C)** 1,25-dihydroxyvitamin D3 concentration **(D)** Ca concentration, and **(E)** P concentration.

There were significant differences in the contents of PTH (*p* < 0.01) and CT (*p* < 0.01) between KB and KN chickens. The PTH content was lower and the CT content was higher in KB chickens (Figures 3A,B **)**. There were no significant differences in serum calcium (*p* > 0.05), serum phosphorus (*p* > 0.05), and 1,25-dihydroxyvitamin D3 (*p* > 0.05) levels between the two groups (Figures 3C–E).

### BMD, BMC, Bone Strength, and Bone Cortical Thickness

**FIGURE 4:**
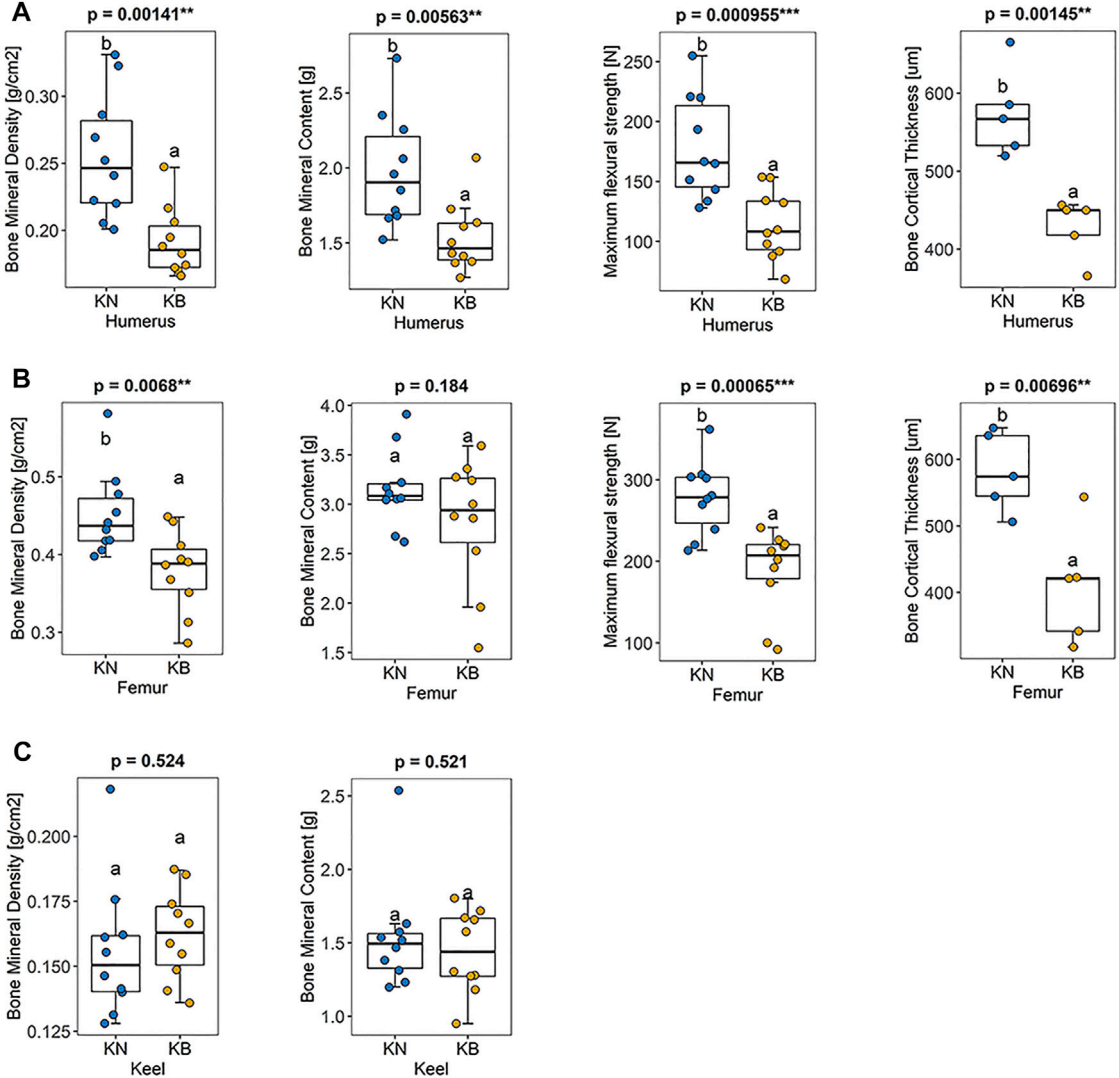
Boxplots of bone mineral density, bone mineral density, bone strength, and bone cortical thickness in KN and KB chicken groups **(A)** Bone index of the humerus **(B)** bone index of the femur, and **(C)** bone index of the keel.

There were significant differences in humeral BMD, humeral BMC, humeral bone strength, and humeral bone cortical thickness between the two groups (*p* < 0.01). These indexes were higher in KN chickens than in KB chickens (Figure 4A). There were significant differences in femoral BMD, femoral humeral bone strength, and femoral bone cortical thickness between the two groups (*p* < 0.01), but there was no significant difference in BMC. For the femur, except BMC, the indexes of KN chicken were higher than those of KB chicken (Figure 4B). The above indexes of KB chickens were poor. There was no significant difference in keel BMC or keel BMD between the two groups (*p* > 0.05; Figure 4C).

### Bone Slice

**FIGURE 5:**
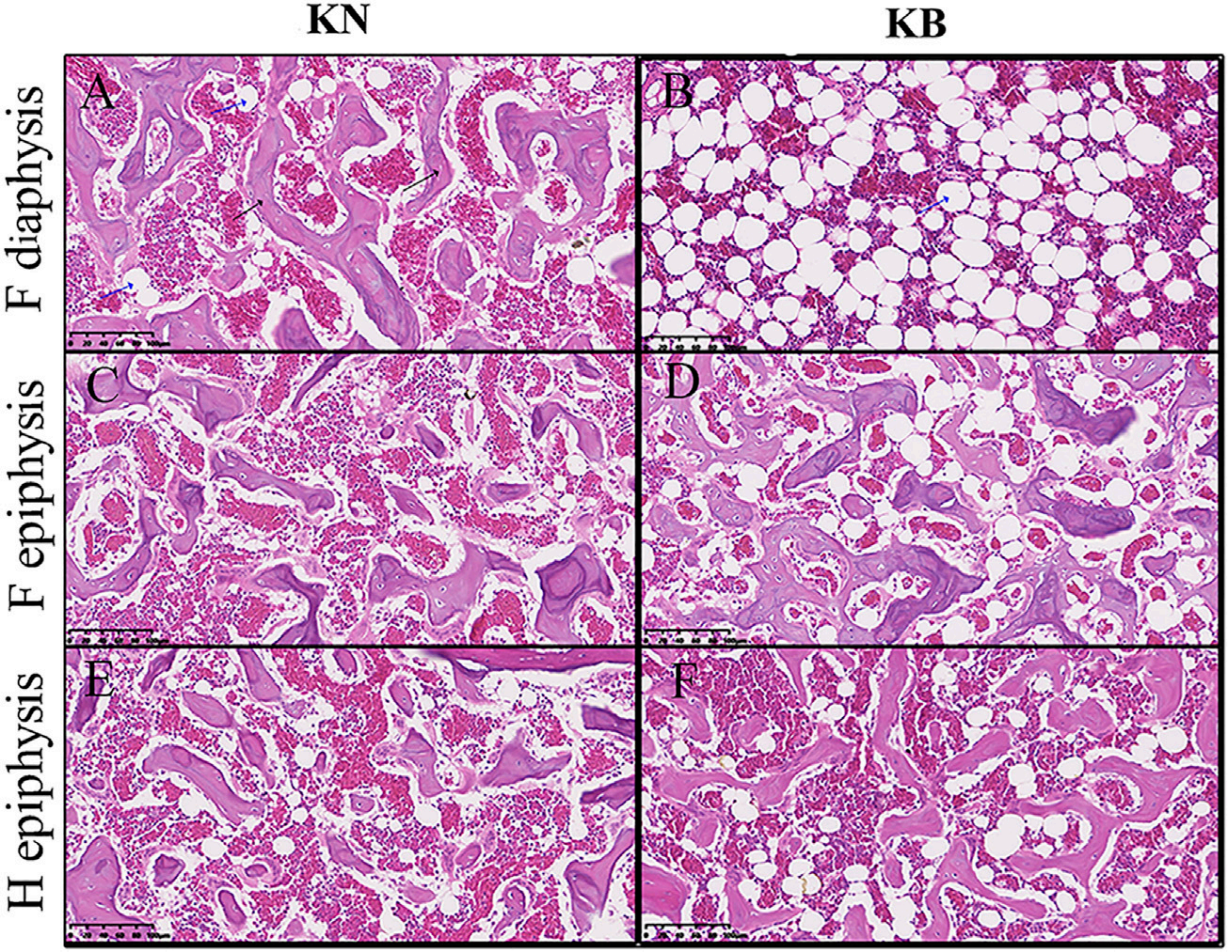
Bone slice chart of the humerus and femur in KN and KB chicken groups. F, femur; H, humerus **(A and B)** Femur diaphysis **(C and D)** femur epiphysis, and **(E and F)** humerus epiphysis. The black arrow indicates the bone trabecular structure. The blue arrow (vacuolated) indicates the adipose tissue.

We sectioned the left humerus and left femur of KB and KN chickens. The diaphysis part was cross cut, and the epiphysis part was longitudinally cut; the difference between the two groups is shown in Figure 5. For the femur, the femoral diaphysis of KN chickens had a bone trabecular structure and a small amount of fat (Figure 5A). The femoral diaphysis of KB chickens had almost no trabecular structure or more adipose tissue (Figure 5B). The femoral epiphysis of both groups had a trabecular bone structure, but the femoral epiphysis of KB chickens contained more adipose tissue (Figures 5C,D). For the humerus, there were no significant differences in humeral diaphysis between the two groups. The humeral epiphysis of the two groups had a trabecular structure, but there was more adipose tissue in the humeral epiphysis of KB chickens (Figures 5E,F).

### Genome Sequencing Data

After quality control, approximately 1,602.40 Mb of clean reads and 480.00 Gb of clean data were obtained, and the Q30 reached an average of 91.76%. The evaluation results of the sequencing output data for each pool are shown in Table 4. The chicken genome was approximately 1.0 GB, with a total of 84 individuals and an average sequencing depth of 5.7x, which meets sequencing requirements. After using the GATK software (v3.6) for mutation detection, the KN group obtained 3.63G data and the KB group obtained 3.76G data.

**TABLE 4:** Evaluation statistics of sequencing data of keel normal (KN) and keel bend (KB) chicken groups.

Sample	KN	KB
Raw_Reads	724,883,017	880,771,276
Clean_Reads	723,196,549	879,203,139
Clean_Bases	216,631,412,100	263,366,444,500
Q20 (%)	96.57	96.74
Q30 (%)	91.57	91.95
GC (%)	42.51	42.49

### Selective Sweep Analysis

**FIGURE 6:**
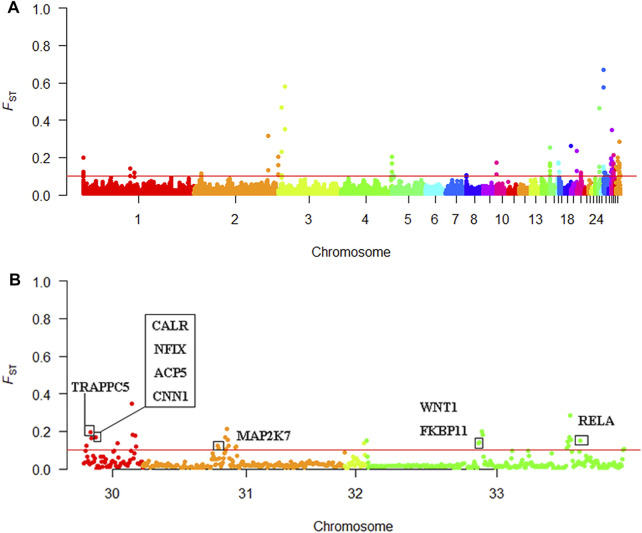
Selective sweep analysis results **(A)** Manhattan plot of the F_ST_ results **(B)** Manhattan plot of chromosomes 30–33.

We screened out 86 regions that may have been affected by selection, as determined by the F_ST_ value (Supplementary Table S1). The mean F_ST_ of the top 1% window was 0.097; therefore, we set the threshold to 0.1. One hundred and twenty-eight genes were discovered using BioMart in the Ensembl database. These genes may be related to KB (Supplementary Table S2). A Manhattan plot of *F*_ST_ results is shown in Figure 6A.

Using Ensembl annotations and based on the function, phenotype description, and previous research reports of these genes, we identified 10 important candidate genes related to keel (Table 5). Among them, nine were located on chromosomes 30, 31, and 33 (Figure 6B), and one was located on chromosome 1. In addition, the PCSK2 found on chromosome three could also be related to bone quality.

**TABLE 5:** List of important candidate genes associated with keel bend.

Chromosome	Gene start (bp)	Gene end (bp)	Window *F*_ST_ value	Gene name
1	930,902	935,155	0.20	ENSGALG00000047166
30	422,263	424,991	0.17	CALR
30	402,432	415,709	0.17	NFIX
30	397,120	399,963	0.17	ACP5
30	392,257	395,386	0.17	CNN1
30	241,789	247,265	0.20	TRAPPC5
30	89,765	111,142	0.12	DNM2
31	2,309,015	2,328,588	0.12	MAP2K7
33	3,422,447	3,427,906	0.14	WNT1
33	3,412,284	3,412,858	0.14	FKBP11
33	6,490,052	6,498,311	0.15	RELA

### GO and KEGG Pathway Enrichment Analysis

**FIGURE 7:**
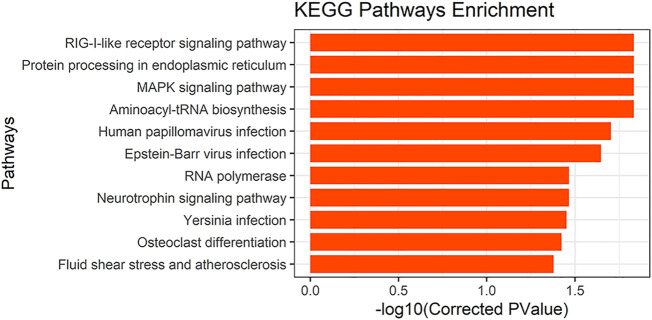
KEGG pathways results.

To determine the possible functions of genes located in the selective scanning region, we converted these gene IDs into human homologous genes to obtain 89 human homologous gene IDs. These were then sent to KOBAS for enrichment analysis (Supplementary Table S2). GO and KEGG pathway analyses were used to determine the biological functions of these genes. We found 11 significantly enriched KEGG pathways (Figure 7). In addition, musculoskeletal diseases and congenital malformations were found in the KEGG disease. Detailed information regarding the analysis of GO, the KEGG pathway, and KEGG disease is shown in Supplementary Table S3.

## Discussion

### Heritability of KB

In this study, we treated KB as a threshold trait by assigning numbers one to four according to the extent of the bend of the keel. A similar strategy has been used in other studies for other factors, such as chicken manure moisture content (Zhu et al., 2020), chicken feather peaking (Brinker et al., 2014), and cow body condition (Edmonson et al., 1989). For the first time, we estimated the heritability of KB. We determined that KB is a heritable trait with medium heritability, indicating that KB is affected by genetic factors. Genetic selection could be an effective method for changing the KB level in the chickens.

We found that the height of the cage was related to the level of KB. The laying hens in the bottom cage showed more severe KB, indicating that the height of the cage is an important factor affecting the level of KB. We speculate that the height of the cage determined many environmental factors, such as air freshness, light intensity, temperature, and humidity. Therefore, raising laying hens in higher cages could be an effective way to reduce KB. At 48 weeks of age, the total number of eggs and the total number of normal eggs of level 4 layers were significantly different from the other three levels, and both were lower. Therefore, our research shows that KB has an impact on the later production performance of laying hens, and severe KB will reduce the production performance of laying hens. Because severe KB usually contain fractures that are not easily detected by palpation. Some results show that normal hens lay more eggs than hens with broken keels (Wei et al., 2020), which is consistent with the results of this study. This may be related to the physiological stress caused by fracture, because fracture will reduce the production performance of laying hens (Mumma et al., 2006).

### Blood Biochemical Indices

PTH and CT play important roles in maintaining calcium and phosphorus balance and regulating bone metabolism. PTH can promote bone resorption and turnover, mobilize bone calcium into the blood, promote the reabsorption of calcium in renal tubules and the absorption of calcium ions in the small intestine, and increase blood calcium and decrease blood phosphorus levels (Goltzman, 2018; Zheng et al., 2020). The main physiological function of CT is to reduce the number of osteoclasts, inhibit the activity of osteoclasts, reduce bone resorption, inhibit the absorption of calcium ions in the small intestine, reduce the concentration of blood calcium *in vivo*, and deposit free calcium in the blood into bone tissue (Naot et al., 2019). There were significant differences in PTH and CT levels between the two groups. The PTH content of KB chickens was lower and the CT content was higher. We speculate that there was relatively little endogenous calcium (calcium in bone) due to the poor bone quality of KB chickens. In order to avoid further loss of endogenous calcium, KB chickens reduce their blood calcium concentration by reducing PTH content and increasing CT content. This maintains a certain level of bone quality while ensuring the amount of calcium required for laying eggs. However, our experimental results showed that there was no significant difference in serum calcium between the two groups, which was different from our prediction. Probably because we are sampling during the day. Because for laying hens, the formation of eggshells mostly occurs at night, and the changes in blood calcium levels may be different at this time. Therefore, the changes of serum calcium levels in laying hens at different times of the day require further study.

### Bone Quality

There were significant differences in almost all bone indices for the humerus and femur of the two groups, with the bone indices of KB chickens being lower than those of KN chickens. The results show that the quality of the bones of the KB chickens was poor, and the keel phenotype may be related to bone quality. This is consistent with previous findings that the fracture strength of the humerus and tibia in chickens with normal keels is greater than that in chickens with abnormal keels (Fleming et al., 2004). However, for the keels of the two groups, there was no significant difference in BMD or BMC, except for the obvious difference in phenotype. Some studies have also reported that there is no difference in keel BMD between individuals with and without fractures (Gebhardt-Henrich et al., 2017). The reasons for this need to be studied further. According to our results, there are significant differences in humerus and femur between KN and KB chickens, but no significant differences in the keel. We believe that the use of endogenous calcium in the different bones of laying hens differs in order to maintain high egg yield. We speculate that laying hens use endogenous calcium in the humerus, femur, and other long bones much more frequently than in the keel. This direction is worthy of in-depth study in the future.

In our study, KB chickens had almost no bone trabecular structure in the femur, which may indicate that KB chickens transfer more medullary bone from the femur for eggshell formation. The bones of hens are mainly composed of cortical, cancellous, and medullary bones. In the long bones (femur, humerus, and tibia, for example), a trabecular structure similar to cancellous bone can be formed periodically, that is, the medullary bone (Dominguez-Gasca et al., 2019). The medullary bone is an unstable intimal bone with low collagen fiber content which mainly exists in the cavity of the long bone (Kerschnitzki et al., 2014). The medullary bone provides an unstable source of calcium for eggshell formation, which calcifies and metabolizes much faster than cortical bone (Whitehead, 2004). The medullary bone is also a unique bone tissue formed only in female birds during the breeding period (Rodriguez-Navarro et al., 2018). It is degraded by the transfer of calcium to the eggshell to supplement the insufficient absorption of calcium and phosphorus in the digestive tract (Hudson et al., 1993).

The femur and humerus of KB chickens contained more adipose tissue, and the BMD of the femur and humerus was also lower. An inverse relationship between BMD and bone marrow adipocyte level has been documented in animal and human studies (Martin and Zissimos, 1991; Sabatakos et al., 2000; Wehrli et al., 2000; Shen et al., 2007; Di Iorgi et al., 2008; Shen et al., 2012b). This relationship has been attributed to the ability of mesenchymal stem cells to differentiate into adipocytes or osteoblasts (Owen and Friedenstein, 1988; Rosen and Bouxsein, 2006). Bone marrow contains different cell populations belonging to several lineages, including hematopoietic stem cells, and mesenchymal stem cells, which can differentiate into osteoblasts, adipocytes, fibroblasts, chondrocytes, and muscle cells (Di Iorgi et al., 2010). Some studies have suggested that the differentiation of bone marrow mesenchymal stem cells into osteoblasts or adipocytes is competitive (Shen et al., 2012a). Similar to other fat pools, bone marrow fat produces adipokines and fatty acids, which might produce a lipotoxic environment in bone cells (Demontiero et al., 2012). Adipocytes inhibit osteoblast proliferation (Maurin et al., 2002) and promote osteoclast differentiation (Hozumi et al., 2009).

### Selective Sweep Analysis

Based on the different phenotypes of keel, we carried out whole-genome Pool-Seq of KN and KB chickens. Selective elimination analysis was then performed to identify genes that could lead to different keel phenotypes. This method has been widely used in SNP and functional gene mining and has achieved good results (Kijas, 2014; Rochus et al., 2018). Our analysis was based on the F_ST_. F_ST_ analysis indicates the degree of population differentiation; the greater the value, the greater the degree of population differentiation and the higher the degree of selection. To narrow the screening range and improve the screening efficiency, we chose *F*_ST_ = 0.1 as the screening threshold. According to gene function annotation and literature, we identified 10 genes with strong selection signals and bone-related genes, namely nine genes with identified functions and one new gene.

*ACP5* encodes tartrate-resistant acid phosphatase (TRACP). TRACP is an abundant protein in osteoclasts, macrophages, and dendritic cells, and its primary substrate is osteopontin (Bilginer et al., 2016). This enzyme is a good marker for bone resorption and osteoclast activity (Bilginer et al., 2016). *ACP5* mutations cause deficient TRACP activity, which results in bone dysplasia through impaired cartilage resorption, particularly at the metaphyses (Lausch et al., 2011). Diseases associated with *ACP5* include spondyloenchondrodysplasia with immune dysregulation (Hayman et al., 1996; Girschick et al., 2015; Briggs et al., 2016; Ramesh et al., 2020). *WNT1* is a member of the Wnt gene family. The Wnt gene family consists of structurally related genes that encode secreted signaling proteins. These proteins have been implicated in oncogenesis and several developmental processes, including regulation of cell fate and patterning during embryogenesis (Zhan et al., 2017). In addition, the Wnt family of proteins drives the development and maintenance of many tissues, including bone (Lu et al., 2018). Numerous human genetic studies and genetically modified mouse models have demonstrated that the Wnt signaling pathway plays an essential role in the regulation of bone formation and resorption (Maeda et al., 2019). *WNT1* plays a role in osteoblast function, bone development, and bone homeostasis (Joeng et al., 2017; Wang et al., 2021). Diseases associated with *WNT1* include osteogenesis imperfecta type Xv and BMD quantitative trait locus 16 (osteoporosis) (Fahiminiya et al., 2013; Keupp et al., 2013; Laine et al., 2013; Pyott et al., 2013; Panigrahi et al., 2018). *CNN1* is a known marker of smooth muscle differentiation, which can bind to actin, tropomyosin, and calmodulin, and is involved in the regulation of smooth muscle contraction activity and cell proliferation (Jiang et al., 1997). It is also expressed in human osteosarcoma cell lines and osteosarcoma tissues (Yamamura et al., 1998). Studies have demonstrated that after knocking out *CNN1*, the degree of ossification in mice increases (Yoshikawa et al., 1998). A recent study found that overexpression of *CNN1* in osteoblasts led to a significant decrease in bone mass at the adult stage by inhibiting osteoblast migration, proliferation, and mineralization, and by promoting osteoclastogenesis (Su et al., 2013). These findings suggest that *CNN1* plays a role in bone-related cells and in the regulation of bone remodeling.

In addition, *NFIX* (Gronostajski, 2000; Adam et al., 2005; Driller et al., 2007; Malan et al., 2010), *CALR* (Klampfl et al., 2013; Nangalia et al., 2013), *FKBP11* (Hanagata et al., 2011; Hanagata and Li, 2011; Tsukamoto et al., 2013), *TRAPPC5* (Sacher et al., 2008; Scrivens et al., 2011), *MAP2K7* (Yamamoto et al., 2002; Svensson et al., 2009), and *RELA* (Frederiksen et al., 2016; Jimi et al., 2019; Yu et al., 2020) have been found to be related to bone health and development in previous studies. Therefore, the above genes are likely to be related to the phenotype of chicken keel, and even affect the development of other bones. Through functional annotation, we identified a new gene related to calmodulin binding (*ENSGALG00000047166*), which is located on chromosome 1. Although this gene has not been reported before, we believe it may be related to bone development. The protein encoded by *PCSK2* is a prohormone processing enzyme involved in insulin and glucagon biosynthesis. Gene polymorphisms are associated with pleiotropic effects on various traits of glucose homeostasis and incident diabetes (Chang et al., 2015). Studies have shown that diabetes is usually accompanied by skeletal fragility (Lecka-Czernik, 2017). Therefore, this gene may indirectly affect bone development.

### Enrichment Analysis

We performed GO ontology and KEGG pathway analyses of the converted human homologous genes. Most genes were enriched in the GO term “protein binding,” and many genes were also enriched in “cytosol,” “nucleoplasm,” “nucleus,” and “cytoplasm” (corrected *p*-value < 0.05). We also found 11 significantly enriched KEGG pathways (corrected *p*-value < 0.05), one of which involves “osteoclast differentiation” and the other is the “MAPK signaling pathway.” A large number of studies have reported that the MAPK signaling pathway is involved in osteoblast differentiation (Chen et al., 2012; Yu et al., 2018; Zhao et al., 2018). In KEGG diseases, we found musculoskeletal diseases and congenital malformations. Musculoskeletal diseases often involve multiple tissues (mainly muscles, bones, cartilage, and nerves; Huang and Sowa, 2011). They are major causes of disability worldwide and have been significant in the development of bone and joint diseases (Brooks, 2006). Congenital malformations are defects in the morphogenesis of organs or body districts that are identifiable at birth (Corsello and Giuffre, 2012). In one study, 3,932 newborns were examined for congenital malformations at birth. Among them, the central nervous system (39.5%) was the most commonly involved, followed by the musculoskeletal system (14.5%; Swain et al., 1994). Through enrichment analysis, We further confirmed that candidate genes screened in this study are likely to be involved in keel development and have an impact on keel phenotype.

## Conclusion

In summary, we found that KB is a medium heritability trait. In hens with obvious KB, the PTH content was lower and the CT content was higher. The bone strength, BMD, and bone cortical thickness of the humerus and femur of hens with obvious KB were lower than those of KN hens, and there was more adipose tissue in the bone. Our results show that the severity of KB is related to bone strength, BMD, and bone cortical thickness. We conducted a selection elimination analysis based on the differences in keel phenotypes between White Leghorn laying hens and identified 10 important candidate genes that have strong selection signals and are related to bones (*ACP5, WNT1, NFIX, CNN1, CALR, FKBP11, TRAPPC5, MAP2K7, RELA, ENSGALG00000047166*). These genes may be related to changes in the keel phenotype of laying hens. The role of other unreported genes requires further research. In the enrichment analysis, two bone-related pathways, the “MAPK signaling pathway” and “osteoclast differentiation”, were enriched, which further verified the reliability of our results. These results may help us to better understand the molecular mechanism of KB in chickens and other birds and provide new insight relevant to the genetic breeding of laying hens.

## Data Availability

The datasets presented in this study can be found in online repositories. The names of the repository/repositories and accession number(s) can be found below: https://www.ncbi.nlm.nih.gov/, PRJNA 772890.
